# Humans’ pupillary contagion extends to cats and dogs

**DOI:** 10.1093/scan/nsaa138

**Published:** 2020-10-07

**Authors:** Emma L Axelsson, Christine Fawcett

**Affiliations:** School of Psychology, The University of Newcastle, Callaghan, 2308, Australia; Department of Psychology, Uppsala University, Uppsala, 751 42, Sweden; Department of Psychology, Uppsala University, Uppsala, 751 42, Sweden

**Keywords:** pupillary contagion, pupillometry, conspecifics, cat person, dog person, pet ownership, empathy

## Abstract

When viewing pupil sizes change, our own pupil sizes change, a phenomenon known as pupillary contagion. This involuntary response is reliable between humans but can be affected by familiarity and empathy. We investigated whether the pupillary contagion response occurs for humans viewing familiar species—cats and dogs—and whether it is modulated by preferences for particular species. Pupil sizes were measured while viewing cat, dog and human images with small, medium and large pupils. Trait empathy, cat and dog affiliation and experience were subsequently measured. There was an image pupil size effect, but this did not vary by species. There was greater pupil size change to cats and dogs than to humans, but this might have been due to the varying size and appearance of the cats and dogs. Greater dog affiliation was also associated with smaller overall pupil size change to dogs and larger change to humans, but this did not interact with image pupil size. Dog affiliation might be associated with less arousal to dog images. In sum, pupillary contagion responses indicate a spontaneous transfer of information about internal states and the findings suggest that humans are sensitive to this across species, regardless of individual preference.

Some behaviours are ‘contagious’ occurring involuntarily after viewing someone else engage in those behaviours (Chartrand and Lakin, 2013). Examples include yawns, smiles and gestures, and numerous studies indicate that they are more likely to happen in response to someone we are familiar with or with whom we have an affiliation (Norscia and Palagi, 2011; Chartrand and Lakin, 2013; Palagi *et al.*, 2014; Mui *et al.*, 2018). Contagious responses can also occur on a physiological level without intention or awareness, including changes in heart rate (Feldman *et al.*, 2011; Helm *et al.*, 2014), neural activity (Anders *et al.*, 2011) and of interest here, pupil size (Laeng *et al.*, 2012; Prochazkova and Kret, 2017). ‘Pupillary contagion’ is a term used to describe the similar change in pupil size when observing someone else’s pupils constrict or dilate (Simms, 1967; Hess, 1975; Harrison *et al.*, 2009). It occurs across the lifespan and emerges early in life as it has been found with infants as young as 4 to 6 months of age (Fawcett *et al.*, 2016, 2017).

Pupillary changes are involuntary responses controlled by the autonomic nervous system (e.g. Steinhauer *et al.*, 2004). Pupils constrict or dilate in response to changes in light exposure, but under stable light conditions, pupil sizes change on the basis of a number of cognitive and emotional factors such as increased working memory load, changes in attention (Hess and Polt, 1964; Bradshaw, 1967; Binda and Murray, 2015), responses to arousing and emotional stimuli (Hess and Polt, 1960; Hess *et al.*, 1965; Partala and Surakka, 2003), when surprised or uncertain (Lavín *et al.*, 2013), and when experiencing aesthetic appreciation of artistic images (Kuchinke *et al.*, 2009). Changes in pupil size can therefore be due to a number of states and to observing stimuli of significance to the observer (Laeng *et al.*, 2012; Prochazkova and Kret, 2017). Pupil dilation is associated with activity in the locus coeruleus in the noradrenergic system, which is associated with allocation of attention (see Laeng *et al.*, 2012 for a review), suggesting it might be involved in attention to significant stimuli.

The pupillary contagion response is based on observing changes in the pupil sizes of others. There are still a number of questions surrounding the pupillary contagion phenomenon and what it reflects, but it is enhanced in adults when observing those from a familiar race and when there is a greater sense of trust in the observer (Kret *et al.*, 2015; Wehebrink *et al.*, 2018). Pupillary contagion is also stronger amongst those who score higher on empathy when viewing highly expressive speakers (Kang and Wheatley, 2017). Providing further evidence for the role of familiarity, Kret *et al.* (2014) found a greater pupillary contagion response in humans and chimpanzees when observing pupillary changes of their own species compared to when observing the other species. The human participants’ pupillary contagion response was larger when viewing pupil size changes in other humans than in chimpanzees and vice versa for the chimpanzees. Prochazkova *et al.* (2018) also found that a network of the brain associated with ‘theory of mind’-related processes is activated particularly when pupils dilate. Therefore, it plays a role in social interactions. Together, the findings on pupillary contagion suggest that it is a non-conscious form of interpersonal synchronization that might serve an important communicative function about internal states of arousal of others and play a role in empathy (Kret *et al.*, 2014; Fawcett *et al.*, 2016).

The aim of the current study was to explore the pupillary contagion response further by examining how it might be affected by the degree of liking, affiliation or familiarity the observer has with the stimuli type. There are no known studies of pupillary contagion responses in humans to cats’ and dogs’ eyes or comparing pupillary contagion responses between human images and familiar animals such as cats and dogs. The specific aim here was to investigate pupillary responses when viewing small, medium and large pupils of cats and dogs as well as humans, not only at a group level but also looking at individual differences for people who identify as so-called ‘cat people’ or ‘dog people’. A ‘cat person’ or ‘dog person’ is defined here as someone who has a particular liking for and affiliation with cats and/or dogs. The terms are often used to indicate that one of the two animals is preferred over the other, but it is also possible to be both or neither (Gosling *et al.*, 2010), and both terms have been used pervasively in English since the 1960s and 70s (‘Google Books Ngram Viewer’, 2013; Google search for ‘cat person’ = 436 000 hits; ‘dog person’ = 422 000).

There are three possible hypotheses for humans’ pupillary contagion responses to cats and dogs. One possibility is that it is a general heightened response to biologically significant stimuli (Amemiya and Ohtomo, 2012) and could be found in response to the pupils of any species with clearly visible pupillary changes. A second possibility is that pupillary contagion will be apparent only, at least to a greater degree, for humans compared to non-humans. This second alternative is supported by the evidence for stronger pupillary contagion effects when observing one’s own species for humans and chimpanzees (Kret *et al.*, 2014). Yet the question remains whether this is because conspecifics are familiar or because they belong to the same species with the same eyes and social properties as the observer. Finally, given the findings that the pupillary contagion response is associated with familiarity, trust and empathy (Kret *et al.*, 2014, 2015; Kang and Wheatley, 2017) and that pupil sizes change when experiencing aesthetic appreciation of artistic images (Kuchinke *et al.*, 2009), a third possibility is that differences in pupillary contagion will be apparent at an individual differences level, such that cat peoples’ pupillary contagion responses to cats’ pupils could be larger than to dogs’, and vice versa for dog people. That is, their greater liking for and familiarity with a species might lead to feelings of warmth, or greater aesthetic appreciation and a likelihood to be more sensitive to any changes in internal states in that species.

If an affiliative sense is important to the pupillary contagion response (e.g. Prochazkova and Kret, 2017), then this might be the case with images of cat and dogs. In a number of Western countries, around 25% of households have a pet cat or dog and they are typically the most common type of pet (US: cats 30%, dogs 37%; UK: cats 26%, dogs 31%; Sweden: cats 17%, dogs 13%; Australia: cats 27%, dogs 38%; Australian Bureau of Statistics, 1995; Murray *et al.*, 2010; American Veterinary Medical Association, 2012; Statistika Centralbyrån, 2012). The statistics are likely higher when taking into account pet ownership over a lifetime. Many regard their pet(s) as important to their lives (see Jacobson and Chang, 2018 for a review), and there is some evidence of psychological and physical health benefits of pet ownership, but the findings tend to conflict (see Saunders *et al.*, 2017 for a review). There is typically a high degree of bonding and affiliation between owners and their pets, which some describe as similar to mother–infant relationships (Nagasawa *et al.*, 2015).

There are no known studies providing objective or physiological evidence that distinguishes cat and dog people or of key interest here, whether there is a greater pupillary contagion response to observers’ preferred animal. There are a number of questionnaire-based measures of bonds between people and their cats and dogs (e.g. Dwyer *et al.*, 2006; Howell *et al.*, 2017). Most studies of cat and dog people are also questionnaire-based personality measures, which have demonstrated, for example, that those identifying as a cat person score higher on neuroticism and openness to experiences, and those identifying as a dog person score higher on extraversion and dominance (Woodward and Bauer, 2007; Gosling *et al.*, 2010; Alba and Haslam, 2015). This suggests that there are potentially differences between people who prefer cats and those who prefer dogs, but there are inconsistencies in the findings across studies and some fail to find any differences between cat and dog people (see Alba and Haslam, 2015 for a review).

A number of studies have looked at physiological responses and neuroendocrine/hormone measures during human–animal interactions (see Beetz *et al.*, 2012 for a review). Studies of human–dog interactions have found increases in oxytocin, endorphin, prolactin, phenylethylamine and dopamine in humans (and dogs), and a decrease in cortisol (Odendaal and Meintjes, 2003; Nagasawa *et al.*, 2015). This has also been found during mutual gaze between humans and dogs (Nagasawa *et al.*, 2015). Curry *et al.* (2015) found that the greater the number of dogs owned over a lifetime, the larger the increase in oxytocin after interactions with dogs, whereas the greater the number of cats owned the greater the reduction in oxytocin after interactions with cats. However, there are inconsistent findings in this area of research too and criticisms surrounding sample sizes and a lack of control conditions (see Beetz *et al.*, 2012; Curry *et al.*, 2015; Powell *et al.*, 2019).

In relation to brain activity, in a human and dog facial recognition task, Shinozaki *et al.* (2007) found different levels of activity in different parts of the anterior cingulate gyrus/cortex (ACC), an area relevant for autonomic functions, attention allocation and social evaluation. One part of the ACC (rostroventral) was responsive during recognition of both humans and dogs, but another part of the ACC (caudal) was responsive only during recognition of human faces. The caudal ACC is associated with more complex evaluation of social stimuli. Despite an affiliation to the dogs, there might be different responses in humans to images of conspecifics than to familiar dogs. This resembles the findings of Kret *et al.* (2014) who found a larger pupillary contagion response to conspecifics for humans and chimpanzees. In the only known study involving responses to cats’ pupils, Amemiya and Ohtomo (2012) found a greater amygdala response in people when viewing cats with larger pupils. This also suggests that pupillary responses to cats’ pupils are plausible, as pupillary responses in humans are associated with activity in the amygdala, which projects to the observer’s brainstem autonomic nuclei, inducing pupillary changes in the observer (Harrison *et al.*, 2006, 2009; Kret *et al.*, 2015).

In the current study, participants were shown a series of images of cats, dogs and humans each with small, medium and large pupil sizes. Changes in pupil sizes of the observers during the presentation of each image were measured as pupillometry is an objective and non-invasive way of measuring physiological responses to stimuli (Binda and Murray, 2015). The aim was to examine not only whether the pupillary contagion phenomenon occurs in humans when viewing other humans as compared to other familiar species but also whether it is influenced by individual differences in how the individual relates to the species being viewed. To assess these individual differences, following the pupillometry measures, participants were asked to rate how much they regard themselves as a cat person, how much they regard themselves as a dog person and, as a measure of familiarity, how many cats and dogs they had had as pets over their lifetime. They also completed questionnaires aimed at measuring their attitudes towards cats and dogs and a questionnaire measuring trait empathy towards people. There are debates surrounding the definition of empathy, but it largely involves a capacity to understand and share another’s emotional state and is an important element of prosocial behaviour (Spreng *et al.*, 2009). Those with higher empathy scores have been found to have stronger pupillary contagion responses (Kang and Wheatley, 2017). Therefore, the pattern of pupil responses from the current study might provide insight into the fundamental properties of the pupillary contagion response. If pupillary contagion is found in response to any animal, then it might best be described as a general heightened response to biologically significant stimuli (Amemiya and Ohtomo, 2012). Alternatively, if a larger pupillary contagion response occurs for humans than for cats and dogs, it would support the idea that pupillary contagion occurs mainly with conspecifics (Kret *et al.*, 2014). However, if the pupillary contagion response is greatest when human observers view their preferred animal, then there is support for pupillary contagion being a reflection of familiarity, trust and empathy (Kret *et al.*, 2014, 2015; Kang and Wheatley, 2017), or even images we find more aesthetically pleasing (Kuchinke *et al.*, 2009). In sum, the findings will address whether the pupillary contagion response is merely an early autonomic response based on properties in the stimuli such as eyes with varying pupil sizes (bottom-up processes) or whether it is also modulated by social biases such as the species and the sense of affiliation to a particular species (top-down processes). The method, research questions and analysis plan for the study were preregistered (https://aspredicted.org/blind.php?x=4zq6c4).

## Method

### Participants

There were 56 participants in the final sample (female = 33, male = 23) with a mean age of 23.86 (*SD* = 5.78; range: 18–46). Participants were recruited from Uppsala University—a large European university in a medium-sized city in Sweden—with flyers and by announcements on class mailing lists. Pupillometry pilot data with a separate sample (*n *= 7) were first collected to test for any unanticipated issues with the images. Another pilot sample (*n *= 11) was used to rate the stimuli (see later in Stimuli subsection).

### Apparatus

Participants were tested using a Tobii Pro Spectrum eye tracker at 120 Hz. The screen measured 52.7 by 29.6cm (23.8 inches diagonal, 1920 × 1080 resolution).

### Stimuli

**Fig. 1.**
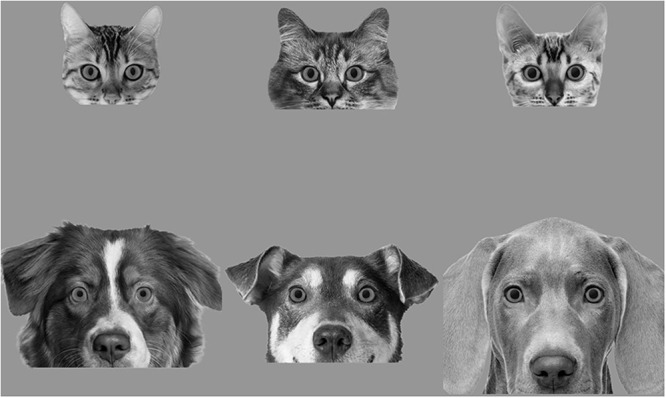
Example cat and dog images with small, medium and large pupils. Surface area of the eyes were equal across species and eye position was in the centre of the screen, and area size of the irises and pupils were the same.

**Fig. 2.**
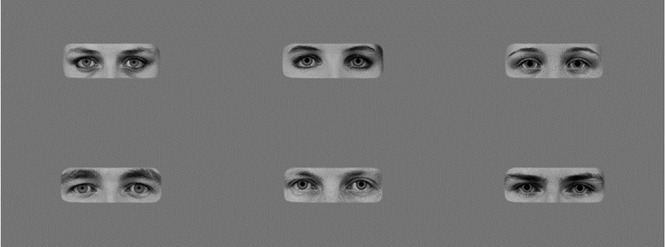
Human eyes, female in top row, male in bottom row with small, medium and large pupils.

In each of the three species presented, cat, dog and human, there were six individuals. Each individual was presented with small, medium and large pupils (18 images per species, see Figures 1 & 2 for example stimuli). All were grey-scaled and presented on a grey background, and all had neutral expressions. As in previous studies (Kret *et al.*, 2015; Fawcett *et al.*, 2017), the large pupils were 40% larger and the small were 40% smaller than the medium. The individual human images had identical irises and pupils, with the pupils differing only in size. The cats and dogs also had identical irises, originally obtained from an image of a cat. The shapes of the pupils differed across cats and dogs, but the colour and area of the small, medium and large pupils was identical across the two species. The dogs’ pupils were round, but the cats’ pupils took on a more vertical shape and the width decreased for the medium and small pupil sizes, which is typical when cats’ pupils contract (Banks *et al.*, 2015).

#### Cat and dog stimuli.

Original images were obtained from Shutterstock.com. The animals that were selected were looking front on to the camera, were symmetrical and there was a clear view of the eyes. As many of the dog images had protruding tongues, the images of both species were cropped from the bottom of the nose for consistency (see Figure 1). The remaining head area was visible to ensure that the species of the animal was clear to participants. Using Adobe Photoshop CS6, all had the backgrounds removed and were placed on a grey background. The final 6 cats and 6 dogs were chosen from a larger set of 21 cats and 17 dogs after obtaining ratings of the original images in relation to attractiveness, gender and emotion from a separate sample of participants (*n *= 11). This was to avoid presenting stimuli which might amplify the pupil sizes for reasons other than pupil size and species type. The aim was to present neutral images and for attractiveness-, gender- or emotion-related characteristics to be similar across the cat and dog images. In relation to gender, participants were asked, ‘Do you think this cat/dog looks masculine or feminine?’ with the following five options: ‘Definitely masculine; Somewhat masculine; Uncertain; Somewhat feminine; Definitely feminine’. In relation to attractiveness, participants were asked, ‘How attractive do you think this cat/dog is?’ with a five-point scale ranging from ‘Not at all’ to ‘Very attractive’. In relation to emotions, participants were asked, ‘Do you think this cat/dog is displaying any of the following emotions?’ The options were: anger, fear, surprise, sadness, disgust, contempt, happiness, ‘none in particular’ or participants could nominate an emotion. To determine the intensity of the emotion perceived, participants were asked, ‘How much emotion do you think the cat/dog is displaying?’ and were presented with a five-point scale ranging from ‘Not much at all’ to ‘A lot of emotion’. Any images with high or low scores for attractiveness, gender and emotion were excluded until the six most neutral and comparable cat and dog images remained (see Table 1 for the ratings for the final six).

**Table 1.** Mean ratings for attractiveness, gender and emotional intensity for the cat and dog images (between 1 and 5)

	Attractiveness		Gender		Emotion	
	*M*	*SD*	*M*	*SD*	*M*	*SD*
Cat 1	3.18	1.08	2.82	0.75	3.18	1.33
Cat 2	3.18	1.08	2.45	0.82	2.45	1.44
Cat 3	3.82	0.98	3.36	1.03	2.91	1.38
Cat 4	3.45	1.13	3.45	0.93	3.09	1.38
Cat 5	3.55	1.04	3.09	1.04	2.73	1.19
Cat 6	3.64	0.81	3.18	0.98	3.27	1.74
Cats Mean	3.47	1.02	3.06	0.93	2.94	1.41
						
Dog 1	3.00	0.78	2.73	1.19	3.64	0.92
Dog 2	3.36	1.03	2.27	1.01	3.09	1.04
Dog 3	4.09	0.70	2.45	1.13	2.55	1.37
Dog 4	3.55	1.04	2.18	0.87	3.09	1.14
Dog 5	2.45	1.21	2.18	0.98	2.18	0.98
Dog 6	3.82	1.08	3.00	0.78	2.64	1.36
Dogs Mean	3.38	0.97	2.47	0.99	2.87	1.14

The images were sized to match for eye size across all animals to ensure that pupil size differences were equally visible across species. This consequently led to differences in head size across cats and dogs (cats: mean width: 15.5 cm, mean height: 13 cm, 14.72˚ × 12.37˚ visual angle at 60 cm distance; dogs: mean width: 29.3 cm, mean height: 19.5 cm, 27.44˚ × 18.46˚ visual angle). Pilot testing revealed possible differences in dilation to the images based on their brightness and size. As it was expected these characteristics could potentially affect pupil size, these were included as covariates in the analyses (see Table 2).

**Table 2.** Size and brightness of images

	Image size			Image brightness	
Species	*M*	*SD*		*M*	*SD*
Cats	30.06	2.76	Small	101.08	3.28
			Medium	98.18	11.69
			Large	97.87	11.69
					
Dogs	92.34	14.04	Small	96.03	27.51
			Medium	95.18	27.64
			Large	94.87	27.64
					
Humans	29.67	0.50	Small	97.41	4.16
			Medium	96.10	0.00
			Large	96.78	4.16

#### Human stimuli.

These were the same as was used in Fawcett *et al.* (2017) and were originally sourced from the Karolinska Directed Emotional Faces (Lundqvist *et al.*, 1998 image IDs: BF19NES, BF13NES, BF01NES, AM14NES, AM10NES and AM08NES). All had neutral expressions and three were male and three female. Only the eyes and surrounding area including eye brows and upper part of nose were visible (see Figure 2 mean width: 23.5 cm, mean height: 8.5cm, 22.16˚ × 8.10˚ visual angle at 60 cm distance).

### Questionnaire measures

Measures of affiliation to cats and dogs as well as trait empathy were included. All questionnaire measures were completed online and were completed directly after pupillometry to avoid any questions from priming responses.

#### Cat and dog affiliation.

This was measured in two ways. One involved asking participants to rate the degree to which they are a ‘cat person’ and the degree to which they are a ‘dog person’ separately with the following question, ‘Which of these statements do you agree with the most?’ Five options were provided, ‘I am not at all a cat/dog person’; ‘I am a little bit of a cat/dog person’; ‘I am somewhat a cat/dog person’; ‘I am very much a cat/dog person’; ‘I am extremely much a cat/dog person’ (translated directly from Swedish). Participants were asked about both cats and dogs instead of asking to them to categorise themselves as either a cat or a dog person to account for people who might be both or neither. This was also due to the findings of Daly and Morton (2006) who found that children who liked and owned both cats and dogs had greater empathy than those who liked or owned only cats or dogs.

The second measure of cat/dog affiliation was the Coleman Dog Attitude Scale (C-DAS; Coleman *et al.*, 2016), which is a measure of general thoughts and feelings about dogs. There was no cat equivalent, so the questionnaire was adapted by substituting the word dog in each question with cat, aside from one, ‘I like to walk dogs’ was changed to a more suitable version for cats, ‘I like to spend time with cats’. There are other questionnaires that measure attitudes to cats, pets or animals, but the C-DAS does not require participants to currently have any pets and asks more general questions (e.g. ‘I love dogs’; ‘When I see a dog I smile’). The measure also correlates moderately with a measure of attitudes to pets, *r* = 0.64 (‘Pet Attitude Scale-Modified’, Munsell *et al.*, 2004). It is also relatively brief (24 items) and has high internal consistency (Cronbach’s α = 0.98) and test–retest reliability (*r* = 0.75).

#### Familiarity with cats and dogs.

This was measured by asking participants how many cats and how many dogs they had had as a pet over their lifetime. This question was included as Curry *et al.* (2015) found a positive relationship between the number of dogs owned over a lifetime and oxytocin levels after interactions with dogs, whereas the opposite relationship was found with cats.

#### Empathy.

The Toronto Empathy Questionnaire is a 16-item questionnaire developed by Spreng *et al.* (2009). It was chosen as it is a brief measure of empathy towards people with a single factor structure measuring largely the emotional more than the cognitive aspects associated with empathy. It has high internal consistency (Cronbach’s α = 0.83–0.85), high test–retest reliability (*r* = 0.81, *p* < 0.001), and high convergent validity correlating well with other measures of empathy (see Spreng *et al.*, 2009).

### Procedure and design

A within groups design was used as all participants saw all species and all three pupil sizes. Images from each species were presented in separate blocks. Given that there is already evidence for a pupillary contagion effect with human images (e.g. Kret *et al.*, 2014), these were always presented in the last block to prevent them from priming responses to the other species. Participants were presented with one of four presentation orders. To counterbalance the appearance of the cat and dog blocks across participants, cats appeared first in two of the orders and dogs in the other two. There were two different pseudo-random orders across the two cat–dog and dog–cat orderings. Within each species block, there were two halves as each image (e.g. Cat1 with small pupils) appeared twice, once in the first half and once in the second half. As there were 6 individuals from each species appearing with each of the 3 pupil sizes, there were 18 images per species. Within a half species block, the 18 images were presented pseudo-randomly with the following caveats: each pupil size was always followed by a different pupil size (i.e. each pupil size never appeared twice in a row); and this was also the case for each individual image (e.g. Cat1 was always followed by a different cat). With each image shown twice, there were 36 trials per species, and a total of 108 trials across the 3 species.

Participants sat approximately 60 cm away from the eye tracker’s screen and were asked to view the images. The experimenter stated that we were interested in their perception of the faces of different species of animals. A standard 5-point calibration was used with the requirement that all points were calibrated before the experiment began. A 1-second grey fixation screen with a small black cross in the centre appeared prior to the presentation of each image. The fixation screen had the same background colour as the images to limit pupillary changes based on the appearance of a new image (Nyström *et al.*, 2015; Fawcett *et al.*, 2016). Each image was presented for 3 seconds. Total presentation time took approximately 7.2 minutes. After pupillometry, the participants completed the questionnaires. Whether participants responded to questions regarding cats or dogs first was counterbalanced across participants.

The main dependent variable (DV) was change in pupil size in response to each image. These pupil size values were calculated in TimeStudio, an open-source, MATLAB-based program for processing time series data (version 3.19; Nyström *et al.*, 2016; timestudioproject.org). Small gaps in the pupil series data of 5 samples or fewer were filled linearly and then the series was smoothed using a moving average of 20 samples. Finally, the pupil size change was calculated for each trial by taking the average of the pupil size during the 3 seconds that the image was presented, minus a baseline of the average pupil size during 0.5 seconds of a fixation screen just before the target image appeared.

## Results

### Exclusion criteria

The following exclusion criteria were set and preregistered prior to running the study: any trials where fewer than 50% of gaze samples were recorded were excluded (*n *= 55 trials; 0.001% of all trials); any trials where the change in pupil size was more than 3 standard deviations from the grand mean for all trials were replaced with the next most extreme pupil value in the data set (*n *= 46 trials; 0.01% of all trials); any participants who did not provide data for at least 2 trials per species and size (e.g. cats with small pupils, and cats with large pupils) were excluded (*n *= 0 participants). On average participants contributed 11.89 trials (of 12, *SD* = 0.42) for each pupil size of a given species and the number of included trials did not differ across the 9 conditions (*M* trial number = 665.89, *SD* = 8.55, χ^2^(4) = 0.46, *p* = 0.978).

### Relationship between cat and dog affiliation variables

Analyses were conducted using jamovi 1.2.22 (jamovi, 2020). Using Spearman’s ρ, the cat person and dog person rating variables were first assessed to determine how they related to the other measures of affiliation to cats and dogs (see Table 3). For cat person rating, there was a significant positive relationship with cat attitude scale and lifetime number of cats owned, and a non-significant negative relationship with dog attitude scale and a significant negative relationship with lifetime number of dogs owned. For dog person rating, there was a significant positive relationship with dog attitude scale and lifetime number of dogs; and a non-significant negative relationship with cat attitude scale and a significant negative relationship with lifetime number of cats. This suggests that the cat and dog person ratings were reflective of their attitudes and experiences with each animal and less so the other. Neither cat nor dog person ratings correlated significantly with empathy. The number of cats owned over a lifetime was significantly greater than the number of dogs, Wilcoxon *W*(55) = 522, *P* = 0.003, *d* = 0.41, and according to the means and medians, this was a difference of 1 (cats *Mdn* = 1; dogs *Mdn* = 0, see Table 3).

**Table 3.** Descriptive statistics for cat/dog affiliation variables and empathy scores and relationship to cat/dog person ratings using Spearman’s rho

	Cat person rating	Dog person rating	Cat attitude scale	Dog attitude scale	Lifetime number of cats	Lifetime number of dogs	Empathy score
*M*	2.95	3.38	81.36	90.86	1.45	0.43	49.43
*SD*	1.18	1.18	26.96	24.13	2.24	0.81	6.28
Cat person rating							
Spearman’s *ρ*			.878	−.016	.525	−.264	.222
*p*			<.001	.204	<.001	.049	.100
Dog person rating							
Spearman’s *ρ*			−.120	.828	−.303	.269	.044
*p*			.377	<.001	.023	.045	.746

### Linear mixed-effects models

The regression analyses were run using linear mixed-effects models in jamovi with the GAMLj module 2.0.1 (Galluci, 2019), which was developed in R (R, 2019) and includes R’s lme4 package (Bates *et al.*, 2015).

#### Preregistered analyses.

There were preregistered analyses, and following feedback during the review process, these are now presented in https://osf.io/ca8tz/. The preregistered analyses involved a backward deletion process starting with a model including all variables, and any non-significant variables were removed sequentially in subsequent models. The complexity of the preregistered analyses might have masked any effects that might be seen with simpler models, thus the main analyses below are now based on simplified models and used a stepwise forward selection process. One outcome of the preregistered analyses was that image brightness and image size were significant in all models and are included in the models presented here. Another outcome of the preregistered analyses is that the initial simple contrasts (in the ‘First Model’) comparing the levels of the image pupil sizes revealed that the change in participant pupil size was significantly smaller for small compared to large image pupils and medium compared to large image pupils. There were also no interactions between image pupil size and species suggesting that the pattern of participant pupil size change in response to the image pupil sizes of all three species was comparable. Therefore, image pupil size was entered as an ordinal variable and polynomial contrasts were performed. This also allowed for more interpretable results in analyses with interactions with empathy and the cat- and dog-related variables.

#### Aggregate of cat and dog person with cat and dog attitude.

In accordance with preregistration and given the strong correlations between the cat person ratings and the cat attitude scores and similarly for the dog-related variables, they were aggregated in IBM SPSS 25 (IBM, 2017). A factor analysis using orthogonal rotation (varimax) was first conducted to determine whether it was possible to aggregate the cat person rating with the cat attitude scale score and dog person rating with the dog attitude scale score. The analysis revealed that there were 2 factors with eigen values over 1, which in combination explained 93.22% of the variance, and the cat person rating and the cat attitude scale score loaded on one factor and the dog person rating and the dog attitude scale score on another (see Tables 4 & 5). These factors were used as variables in the subsequent analyses and have been relabelled as ‘cat person/attitude’ and ‘dog person/attitude’.

**Table 4.** Initial factor analysis with number of components and total variance explained

	Initial eigenvalues			Extraction sums of squared loadings	
Component	Total	% of Variance	Cumulative %	Total	% of Variance
1	2.06	51.53	51.53	2.06	51.53
2	1.67	41.70	93.22	1.67	41.70
3	.18	4.40	97.62		
4	.10	2.38	100.00		

**Table 5.** Factor analysis with total variance explained following varimax rotation

Component	Extraction sums of squared loadings	Rotation sums of squared loadings			Factor loadings	
	Cumulative %	Total	% of Variance	Cumulative %		
1	51.53	1.87	46.64	46.64	Cat Person Rating Cat Attitude Score	.96 .97
2	93.22	1.86	46.58	93.22	Dog Person Rating Dog Attitude Score	.96 .97
3						
4						

#### Main analyses.

The analyses involved a stepwise forward selection process to enable the ability to determine the effects of image pupil size and species on the DV, change in participant pupil size, as well as the effects of empathy and the cat and dog affiliation and experience variables. The effects of the variables were assessed within models, but comparisons between models were also performed with likelihood ratio tests (LRT) based on the models’ –2 log-likelihood (−2LL) values. A key model contained image pupil size and species (Model 6) and this was the main comparison model for the subsequent additions of empathy and the cat and dog affiliation and experience variables. Specifically, a preliminary collection of models (Set 0) involved a null model with the random effects of participant and the low-level image feature covariates. The first collection of models (Set 1) involved assessing the main effect of image pupil size on the DV, to determine if there was an overall pupillary contagion effect followed by the effects of species and the interaction with image pupil size to see if the response to the image pupil sizes differed across the three species. The following five sets of models tested whether there were effects of the individual differences in pupillary contagion. That is, they involved adding each individual difference variable and their interactions to see if they interacted with the image pupil sizes of each species: empathy (Set 2), cat person/attitude (Set 3), dog person/attitude (Set 4), lifetime number of cats (Set 5) and lifetime number of dogs (Set 6). Aside from assessing the contribution of each variable within a model, model fit was also compared to Model 6 (containing image pupil size and species) to determine the effect of introducing each individual difference variable. In all models, maximum likelihood was used to estimate the parameters as we were largely interested in the fixed effects. The default convergence optimiser (bobyqa) was used. For the tests of the fixed effects, the default Type III ANOVA-style *F*-tests were used and degrees of freedom were calculated using the Satterthwaite method.

#### Set 0: null model and low-level image feature covariates.

Model 1 included participant only as a random effect and was significant within the model, LRT(1) = 124.41, *P* < 0.001 (see also Table S2 in Supplementary materials). Model 2 involved the addition of image size, which was significant within the model, *F*(15,938.20) = 141.61, *P* < 0.001, and compared to the null model (see Tables S1 and S3 in Supplementary Materials). Image brightness was further added revealing that it was also significant within the model, *F*(15,938.25) = 3182.49, *P* < 0.001, and compared to Model 2 (see Tables S1 and S4 in Supplementary Materials). Therefore, image size and brightness likely had an impact on change in participant pupil size and were retained in subsequent models.

### Was there pupillary contagion across species?

#### Set 1: image pupil size and species.

**Fig. 3.**
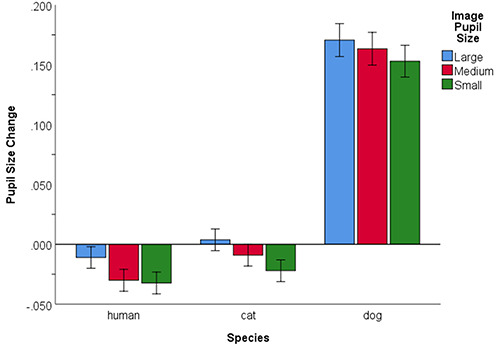
Pupil size (means and standard error) as a function of species of pupil image sizes.

In Model 4, image pupil size was added to Model 3 to test for an overall pupillary contagion effect. Image pupil size was significant within the model, *F*(25,937.17) = 6.65, *P* < 0.001, and compared to Model 3 (see Table S1 in Supplementary Materials). Polynomial contrasts revealed that there was also a significant linear effect of image pupil size with participants’ pupillary size changing relative to the pupil sizes in the images (large: *M* = 0.054, *SE* = 0.007; medium *M* = 0.041, *SE* = 0.007, small *M* = 0.034, *SE* = 0.007; see also Table S5 in Supplementary Materials). Model 5 involved the addition of species, which was significant within the model, *F*(25,938.51) = 55.63, *P* < 0.001, and compared to the previous model (see Table S1 in Supplementary Materials). Simple contrasts revealed that there was also a significantly smaller change in participant pupil size in response to the human (*M* = −0.025, *SE* = 0.009) compared to the cat pupils (*M* = −0.009, *SE* = 0.009) and compared to the dog pupils (*M* = 0.162, *SE* = 0.013, see Table S6 in Supplementary Materials). In Model 6, the interaction between image pupil size and species was added revealing that it was non-significant within the model, *F*(45,937.21) = 0.29, *p* = 0.882, and compared to Model 5 (see Table S1 in Supplementary Materials and Table 6 below). This suggests that there was an overall pupillary contagion effect that did not differ significantly across the three species (see Figure 3). To assess the role of participant sex, this was added to Model 6, first on its own (Model 7) and as an interaction with image pupil size (Model 8). These were non-significant within the models and in the comparisons between Models 7 and 6 and Models 8 and 7 (see Table S1 in Supplementary Materials). Finally, the random slopes for image pupil size and species were each added in separate models (Models 6.1 & 6.2), but this led to a singular model fit in each. Therefore, the subsequent models were compared to Model 6.

**Table 6.** Model 6’s fixed effect parameter estimates and random effect variance and correlation values

*Fixed effects parameter estimates*								95% Confidence interval					
Names	Effect			Estimate		*SE*		Lower		Upper	*df*	*t*	*P*
(Intercept)	(Intercept)			0.043		0.006		0.031		0.055	56.053	6.870	< .001
Image_size	Image_size			−0.002		2.731e−4		−0.002		−0.001	5939.445	−5.902	< .001
Image_brightness	Image_brightness			−0.008		1.479e−4		−0.008		−0.007	5937.553	−51.742	< .001
Image_pupil_size1	Linear			−0.015		0.004		−0.023		−0.008	5937.168	−3.955	< .001
Image_pupil_size2	Quadratic			0.002		0.004		−0.006		0.009	5937.167	0.479	0.632
Species1	Cat - human			0.016		0.005		0.005		0.026	5937.876	2.827	0.005
Species2	Dog - human			0.187		0.018		0.152		0.222	5939.165	10.455	< .001
Image_pupil_size1 ✻ species1	Linear ✻ cat - human			−0.003		0.009		−0.022		0.015	5937.142	−0.342	0.732
Image_pupil_size2 ✻ species1	Quadratic ✻ cat - human			−0.007		0.010		−0.026		0.012	5937.293	−0.722	0.470
Image_pupil_size1 ✻ species2	Linear ✻ dog - human			0.002		0.010		−0.016		0.021	5937.189	0.253	0.800
Image_pupil_size2 ✻ species2	Quadratic ✻ dog - human			−0.008		0.010		−0.027		0.011	5937.414	−0.834	0.404
*Random components*		Groups	Name		*SD*	Variance	*ICC*						
		Participant	(Intercept)		0.044	0.002	0.059						
		Residual			0.173	0.030							

### Does pupillary contagion interact with individual differences in empathy?

#### Set 2: image pupil size, species and empathy.

To test for the role of empathy in change in participant pupil size, empathy was added to Model 6 first on its own (Model 9), followed by the additions of the following interactions: empathy by image pupil size (Model 10), empathy by species (Model 11), empathy by image pupil size and empathy by species (Model 12) and empathy by image pupil size by species (Model 13). In all models, the effects were non-significant and the comparisons between Models 9 and 6 and comparisons between succeeding models were non-significant (see Table S1 in Supplementary Materials). This suggests that empathy scores had little relation to participants’ responses to the images.

### Does pupillary contagion interact with an affiliation to cats or dogs?

#### Set 3: image pupil size, species, and cat person/attitude.

To test for the role of an affiliation to cats, cat person/attitude (aggregate of cat person rating and cat attitude score) was first added to Model 6 on its own (Model 14), followed by the additions of the following interactions: cat person/attitude by image pupil size (Model 15), cat person/attitude by species (Model 16), cat person/attitude by image pupil size and cat person/attitude by species (Model 17), and cat person/attitude by image pupil size by species (Model 18). In all models, the effects were non-significant and the comparisons between Models 14 and 6 and comparisons between succeeding models were non-significant (see Table S1 in Supplementary Materials). This suggests that cat person/attitude scores had little relation to participants’ responses to the images.

#### Set 4: image pupil size, species, and dog person/attitude.

**Fig. 4.**
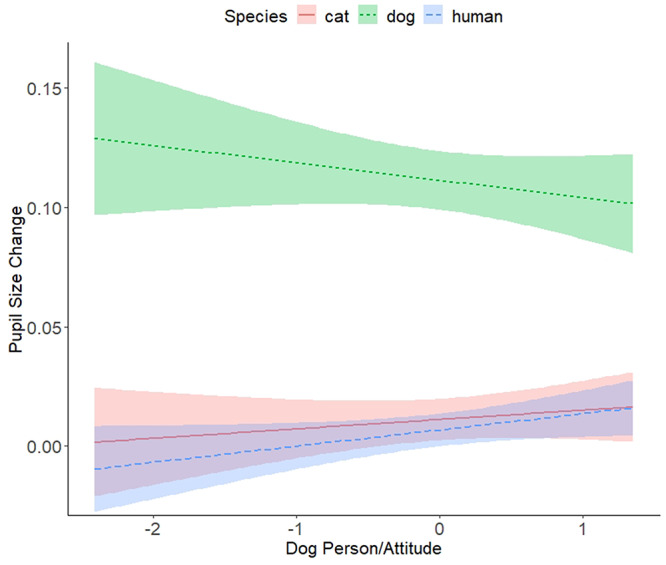
Pupil size change as a function of dog person/attitude and species of pupil image sizes.

To test for the role of an affiliation to dogs, dog person/attitude (aggregate of dog person rating and dog attitude score) was first added to Model 6 on its own (Model 19), followed by the additions of the following interactions: dog person/attitude by image pupil size (Model 20), dog person/attitude by species (Model 21), dog person/attitude by image pupil size and dog person/attitude by species (Model 22), and dog person/attitude by image pupil size by species (Model 23). The comparison between Model 19 and 6 was non-significant, but there was a significant difference between Models 21 and 19 following the addition of the interaction between dog person/attitude and species. Within Model 21, the dog person/attitude by species interaction was significant, *F*(25,937.40) = 4.31, *p* = 0.014. The tests of the fixed effect estimates revealed that dog person/attitude interacted differently between responses to human and dog pupils (see Table 7). The greater the dog affiliation score the smaller the overall pupil size change to dog images, which contrasted with a larger overall pupil size change to human images (see also Figure 4). All other comparisons between the models were non-significant (see Table S1 in Supplementary Materials).

**Table 7.** Model 21’s fixed effect parameter estimates and random effect variance and correlation values

*Fixed effects parameter estimates*				95% Confidence interval				
Names	Effect	Estimate	*SE*	Lower	Upper	*df*	*t*	*P*
(Intercept)	(Intercept)	0.043	0.006	0.031	0.055	56.054	6.870	< .001
Image_size	Image_size	−0.002	2.729e−4	−0.002	−0.001	5939.432	−5.917	< .001
Image_brightness	Image_brightness	−0.008	1.478e−4	−0.008	−0.007	5937.550	−51.787	< .001
Image_pupil_size1	Linear	−0.015	0.004	−0.023	−0.008	5937.169	−3.955	< .001
Image_pupil_size2	Quadratic	0.002	0.004	−0.006	0.009	5937.167	0.479	0.632
Species1	Cat - human	0.016	0.005	0.005	0.026	5937.876	2.829	0.005
Species2	Dog - human	0.187	0.018	0.152	0.222	5939.155	10.472	< .001
Dog_person/attitude	Dog_person/attitude	8.744e−4	0.006	−0.011	0.013	55.995	0.140	0.889
Image_pupil_size1 ✻ species1	Linear ✻ cat - human	−0.003	0.009	−0.022	0.015	5937.142	−0.342	0.733
Image_pupil_size2 ✻ species1	Quadratic ✻ cat - human	−0.007	0.010	−0.026	0.012	5937.293	−0.722	0.470
Image_pupil_size1 ✻ species2	Linear ✻ dog - human	0.002	0.009	−0.016	0.021	5937.188	0.255	0.799
Image_pupil_size2 ✻ species2	Quadratic ✻ dog - human	−0.008	0.010	−0.027	0.011	5937.414	−0.837	0.403
Species1 ✻ dog_person/attitude	Cat - human ✻ dog_person/attitude	−0.003	0.005	−0.014	0.008	5937.462	−0.536	0.592
Species2 ✻ dog_person/attitude	Dog - human ✻ dog_person/attitude	−0.015	0.005	−0.026	−0.004	5937.510	−2.766	0.006
*Random components*	Groups	Name	*SD*	Variance	*ICC*			
	Participant	(Intercept)	0.044	0.002	0.059			
	Residual		0.173	0.030				

### Does pupillary contagion interact with experience with cats or dogs?

#### Set 5: image pupil size, species and lifetime number of pet cats.

To test for the role of experience with cats on pupil size change, the participants’ number of pet cats owned over their lifetime was added to the model. Due to issues with skewness, this variable was square root transformed. It was first added to Model 6 on its own (Model 24), followed by the additions of the following interactions: number of cats by image pupil size (Model 25), number of cats by species (Model 26), number of cats by image pupil size and number of cats by species (Model 27), and number of cats by image pupil size by species (Model 28). In all models, the effects were non-significant and the comparison between Models 6 and 24 and comparisons between succeeding models were non-significant (see Table S1 in Supplementary Materials). This suggests that the lifetime number of pet cats owned had little relation to the pupillary changes.

#### Set 6: image pupil size, species and lifetime number of pet dogs.

To test for the role of experience with dogs on pupil size change, the participants’ number of pet dogs owned over their lifetime was added to Model 6 in Model 29. This variable was also square root transformed due to skewness. The following interactions were also added: number of dogs by image pupil size (Model 30), number of dogs by species (Model 31), number of dogs by image pupil size and number of dogs by species (Model 32), and number of dogs by image pupil size by species (Model 33). In all models, the effects were non-significant and the comparison between Models 6 and 29 and comparisons between succeeding models were non-significant (see Table S1 in Supplementary Materials). This suggests that the lifetime number of pet dogs owned also had little relation to the pupillary changes.

## Discussion

In accordance with the preregistered hypotheses and research questions for this study, we expected to replicate previous studies with human participants showing greater pupillary change to images of other humans with dilated pupils (Hess, 1975; Harrison *et al.*, 2009; Fawcett *et al.*, 2017). However, the main aim was to test whether humans show pupillary contagion differentially to other humans as compared to non-humans as found by Kret *et al.* (2014). A key question here was also whether there would be an effect of viewing varying pupil sizes of non-humans from a familiar species, and whether this contagion tendency is related to individual difference characteristics such as empathy, affiliation with cats and dogs, or experience with cats and dogs. Linear mixed-effects models were used to address the above questions. While our initial preregistered analyses are now presented in https://osf.io/ca8tz/ and the new analyses are presented in the manuscript and supplementary materials, the effects found in both sets of analyses were largely the same.

The first set of models (Set 1) demonstrated an effect of image pupil size such that the changes in participant pupil sizes corresponded to the image pupil sizes. The descriptive statistics and linear trend indicated that the change was smaller in response to small image pupils compared to large. With the addition of species, the model fit improved. There was an effect of species such that the change in pupil size was smaller to humans overall relative to the cat and the dog images. It is unclear if this is due to a conspecific relative to non-human effect, but it is more likely due to greater variability in the cat and dog images. Importantly, there was no interaction between image pupil size and species, showing that the contagion effect did not differ across species. The findings suggest that the image pupil sizes affected participant pupil size regardless of the species. In subsequent sets of models, the individual difference variables, empathy, cat person/attitude, dog person/attitude, lifetime of number of cats and dogs were non-significant and did not interact with image pupil size and species. However, there was a species by dog person/attitude interaction such that greater dog affiliation scores were associated with smaller overall pupil size changes to dog images and larger pupil size changes to humans. Therefore, those with a greater ‘dog affiliation’ are perhaps less ‘startled’ by the variation in dog images. However, the individual difference variables did not interact with image pupil size and species. This was also seen in the preregistered analyses (see https://osf.io/ca8tz/). The findings suggest that pupillary responses can occur in response to any species with visible pupils regardless of any affiliation with a particular species.

Therefore, in relation to the first research question, the expected pupillary contagion response seen in previous studies (Hess, 1975; Harrison *et al.*, 2009; Fawcett *et al.*, 2017) was seen here with human participants’ pupil sizes changing based on the observed human pupil sizes. In relation to the second preregistered research question, there did not appear to be a conspecific effect. Our results revealed a general effect of image pupil size regardless of the species being viewed, supporting the view that the pupillary contagion response might be a general heightened response to biologically significant stimuli (Amemiya and Ohtomo, 2012). This suggests that the pupillary contagion response could be found in response to any animal with clearly visible pupillary changes. However, when taking into consideration the findings from Kret *et al.* (2014) where there was a larger pupillary contagion response in humans and chimpanzees in response to conspecifics than to the contrasting species, our results may actually point to a general familiarity effect where humans need to be at least somewhat familiar with or have experience with a species for them to respond to that species with pupillary contagion.

While there was no difference in pupillary contagion across species, there was a significant main effect of species such that the overall pupillary response was smallest to human images and larger to cats and in particular dogs (see Figure 3). While this could be due to features of the images, we did adjust for image size and brightness in our analyses. Still, the dogs were the most variable in these perceptual features, so it is possible that during the blocks of dog images, the changes in size and brightness from image to image resulted in greater cognitive processing and thus greater pupil size change.

The remaining sets of models addressed the third and fourth preregistered research questions by examining the effect of individual differences in empathy (Set 2), cat person/attitude (Set 3), dog person/attitude (Set 4), lifetime number of cats (Set 5) and lifetime number of dogs (Set 6) on pupillary contagion to the different species. These questions were raised due to the findings that the pupillary contagion response is a reflection of familiarity, trust and empathy (Kret *et al.*, 2014, 2015; Kang and Wheatley, 2017) and that pupil dilation can occur for images that are aesthetically pleasing (Kuchinke *et al.*, 2009). It was assumed that those who have a greater affinity towards cats over dogs or vice versa might have a greater degree of familiarity, feelings of warmth or greater aesthetic appreciation with the species and perhaps be more sensitive to any changes in internal states in their preferred species. Aside from the smaller pupil size changes to dogs relative to humans in those with greater dog affiliation, there were no significant effects of the individual difference variables on overall pupil size change or on pupillary contagion. This indicates that the pupillary contagion effects are consistent both across species and across individual factors concerning how a person feels about and relates to that species. Together, our results do not support that individual differences in affiliation to particular species enhance pupillary contagion responses.

Previous findings that the pupillary contagion response is a reflection of familiarity, trust and empathy (Kret *et al.*, 2014, 2015; Kang and Wheatley, 2017) are based on studies with humans responding to human pupils and therefore the effect might not be applicable to humans responding to non-human animal images. On the other hand, a lack of an expected cat person or dog person effect per se and only a general image pupil size effect might suggest that people are sensitive to the internal states of all species. The ability to be perceptive to the internal states of other species might be important in interactions with other species in general and could even be important in discriminating predators and prey (Barrett, 2005). This might be useful in determining not only the internal states but also in predicting the behaviour of other animals, regardless of a preference or affiliation for a particular species.

The study has, however, revealed a previously unknown pupillary contagion response in humans to cats’ and dogs’ pupils. This preregistered study was well powered and the lack of a cat or dog person individual difference effect is unlikely due to an insufficient sample size. The stimuli were high-quality images that were carefully constructed to appear as natural as possible. The analyses also allowed for the effect of differences in image size and brightness to be adjusted for. There were, however, a few potential weaknesses with the study. Despite the size of the images being adjusted for, the larger and more variable dog images did seem to affect pupil responses, making it more difficult to determine if there is a conspecific effect or not. When creating the stimuli, we aimed to have equally sized eyes across the species and a representative sample of members of each species, which then led to different sized heads across species as the ratio of eye to head size differs across cats, dogs and humans. While this diversity makes the stimuli more ecologically valid, future studies could use more controlled stimuli by perhaps only showing the same-sized eye region of all three species, with a limited range of species so that size and brightness can be matched across images, and inform participants beforehand which species they are viewing. Another issue is that presenting animated rather than static pupils could be even more realistic and thus might provide a more sensitive measure of the pupillary contagion response.

The final cat and dog images were selected from a larger set based on ratings on attractiveness, gender, and emotion from a separate sample of participants. Any images with extreme scores were removed to limit pupillary changes occurring for reasons other than image pupil size. However, the final set of images then had the new irises and pupils added. For future studies, ratings for these should also have been obtained. In particular, perceptions of attractiveness have been implicated in pupillary responses (Kuchinke *et al.*, 2009; Kret and De Dreu, 2019). We had aimed in this study to limit the influence of attractiveness, by removing animals that were rated as too attractive/unattractive and kept the images that had similar attractiveness ratings (cats: *M* = 3.47, *SD* = 0.26; dogs: *M* = 3.38, *SD* = 0.59, *t*(5) = 0.43, *P* = 0.683, *d* = 0.18). Regardless, those with a stronger affiliation to one of the species might have perceived them as more attractive. However, this is unlikely as there was no clear interaction between image pupil size and of cat/dog affiliation. Nonetheless, participants’ ratings of the stimuli could interact with their pupillary responses and should be measured.

Another issue is that only relatively familiar species were tested as cats and dogs are typically the most popular type of pet (Australian Bureau of Statistics, 1995; Murray *et al.*, 2010; American Veterinary Medical Association, 2012; Statistika Centralbyrån, 2012). Aside from the findings of Kret *et al.* (2014) with chimpanzees, it is unknown whether a pupillary contagion effect would occur with less familiar species. Future studies should compare responses to more typically and less typically encountered species to determine whether it is general heightened response to biological images or only familiar species. Future studies should also present images of individual animals that are known to the participants (e.g. their own pet). The lack of a clear cat or dog person effect could be due to the use of unfamiliar cat and dog images raising questions as to whether known animals or more familiar or preferred breeds would lead to a stronger effect.

Another issue is that the numbers of cats and dogs owned over a lifetime was quite low with the current sample. Curry *et al.* (2015) found that the greater the number of dogs owned over a lifetime, the larger the increase in oxytocin after interactions with dogs, whereas the greater the number of cats owned the greater the reduction in oxytocin after interactions with cats. Finding a sample of participants with greater experience with cats and dogs might be necessary to uncover individual differences based on this factor. Diamond and Carey (1986) found a face inversion effect in dog experts to dog faces and given that inversion effects are typically associated with familiarity, what would the pupillary effect be for those who spend a large degree of time with pets or with a particular species?

The split between female (*n* = 33) and male participants (*n* = 23) was not equal. Although this difference was non-significant, χ^2^(1) = 1.77, *P* = 0.181, and the effect of participant sex was non-significant, there is a possibility that a greater proportion of one sex could have had an effect on the results. For example, Schirmer *et al.* (2013) found that female participants were faster and more accurate in emotion recognition tasks with human and dog faces. This was particularly the case in an implicit emotional valence priming task. The emotional expressions in the faces presented here were neutral, but if there is an effect of participant sex in responses to animal faces, the male/female split should be comparable in future studies.

Pupillometry is an objective and non-invasive way of measuring physiological responses to stimuli (Binda and Murray, 2015). The findings here suggest that the pupillary contagion response is an early autonomic response based on properties in the stimuli such as eyes with varying pupil sizes of any species (bottom-up processes). However, the evidence here did not clearly indicate that it was modulated by social biases (top-down processes) such as cat and dog affiliation. The findings did, however, indicate that the pupillary contagion response does occur to animal images and in particular, animals that are the most common type of pets. Pets are important to many people, and research into the relationships between people and their pets can provide a greater understanding of the complexities underlying these relationships and it might contribute to the understanding of people’s perception and treatment of animals (Alba and Haslam, 2015; Su *et al.*, 2018). A recent study has revealed that people are sensitive to cats’ affective states from subtle facial cues (Dawson *et al.*, 2019). The findings here suggest that people might also be perceptive to the internal states of familiar species such as cat and dogs via changes in pupil size.

## Supplementary Material

nsaa138_Supp
