# Supplementary material for: Humans’ pupillary contagion extends to cats and dogs
Source: Soc Cogn Affect Neurosci. 2020 Oct 7;16(1-2):153–66. doi: 10.1093/scan/nsaa138 (PMC7812621; doi:10.1093/scan/nsaa138)
Supplement: nsaa138_Supp [file nsaa138_supp.zip › scan-19-432-File013.docx]

Table of Contents

[TABLE A1 2](#_Toc49883687)

[*COMPLETE LIST OF MODELS AND COMPARISONS BETWEEN MODELS* 2](#_Toc49883688)

[Set 0: null model and stimuli covariates 2](#_Toc49883689)

[Set 1: addition of image pupil size and species 2](#_Toc49883690)

[Set 2: addition of empathy to M6 3](#_Toc49883691)

[Set 3: addition of cat person/attitude to M6 4](#_Toc49883692)

[Set 4: addition of dog person/attitude to M6 5](#_Toc49883693)

[Set 5: addition of lifetime number of pet cats (square root) to M6 6](#_Toc49883694)

[Set 6: addition of lifetime number of pet dogs (square root) to M6 7](#_Toc49883695)

[TABLE B1 8](#_Toc49883696)

[*MODEL 1’S (NULL MODEL) FIXED EFFECT PARAMETER ESTIMATES AND RANDOM EFFECT VARIANCE AND CORRELATION VALUES* 8](#_Toc49883697)

[TABLE B2 8](#_Toc49883698)

[*MODEL 2’S FIXED EFFECT PARAMETER ESTIMATES AND RANDOM EFFECT VARIANCE AND CORRELATION VALUES* 8](#_Toc49883699)

[TABLE B3 9](#_Toc49883700)

[*MODEL 3’S FIXED EFFECT PARAMETER ESTIMATES AND RANDOM EFFECT VARIANCE AND CORRELATION VALUES* 9](#_Toc49883701)

[TABLE C1 10](#_Toc49883702)

[*MODEL 4’S FIXED EFFECT PARAMETER ESTIMATES AND RANDOM EFFECT VARIANCE AND CORRELATION VALUES* 10](#_Toc49883703)

[TABLE C2 11](#_Toc49883704)

[*MODEL 5’S FIXED EFFECT PARAMETER ESTIMATES AND RANDOM EFFECT VARIANCE AND CORRELATION VALUES* 11](#_Toc49883705)

# TABLE A1

# *COMPLETE LIST OF MODELS AND COMPARISONS BETWEEN MODELS*

| **Model specification** | **Model**  **name** | **Fixed Effects** | **Fixed Effects added** | **Random Effects** | | **Model fit** | | | | **LRT Test** | | |
| --- | --- | --- | --- | --- | --- | --- | --- | --- | --- | --- | --- | --- |
|  |  |  |  | **intercept** | **slope** | ***AIC*** | ***BIC*** | ***-2LL*** | ***df*** | **Comparison** | ***df*** | **X^2^** |
| Set 0: null model and stimuli covariates | | | | | | | | | | | | |
| RE only | M1 |  | - | participant |  | -1064.73 | -1044.63 | -1070.73 | 3 |  |  |  |
| Covariate | M2 | image size | image size | participant |  | -1202.68 | -1175.88 | -1210.68 | 4 | M2 vs M1 | 1 | -139.95* |
| Covariates | M3 | image size +  image brightness | image brightness | participant |  | -3749.10 | -3715.61 | -3759.10 | 5 | M3 vs M2 | 1 | -2548.42* |
| Set 1: addition of image pupil size and species | | | | | | | | | | | | |
| FE main effect + covariates | M4 | image size +  image brightness +  image pupil size | image pupil size | participant |  | -3758.39 | -3711.50 | -3772.39 | 7 | M4 vs M3 | 2 | -13.30* |
| FE main effects + covariates | M5 | image size +  image brightness +  image pupil size +  species | species | participant |  | -3864.62 | -3804.34 | -3882.62 | 9 | M5 vs M4 | 2 | -110.22* |
| FE main effects + 2-way interaction + covariates | M6 | image size +  image brightness +  image pupil size +  species +  species × image pupil size | species × image pupil size | participant |  | -3857.80 | -3770.72 | -3883.80 | 13 | M6 vs M5 | 2 | -1.18 |
| FE main effects + 2-way interaction + covariates | M7 | image size +  image brightness +  image pupil size +  species +  species × image pupil size +  participant sex | participant sex | participant |  | -3857.60 | -3763.82 | -3885.60 | 14 | M6.1 vs M6 | 1 | -1.80 |
| FE main effects + 2-way interaction + covariates | M8 | image size +  image brightness +  image pupil size +  species +  species × image pupil size +  participant sex +  participant sex × image pupil size | participant sex × image pupil size | participant |  | -3854.20 | -3747.03 | -3886.20 | 16 | M6.2 vs M6.1 | 2 | -0.60 |
| FE main effect + slope + covariates | M6.1 | image size +  image brightness +  image pupil size +  slope of image pupil size | slope of image pupil size | participant | image pupil size | Model fit was singular | | | |  |  |  |
| FE main effects + slope + covariates | M6.2 | image size +  image brightness +  image pupil size +  species +  slope of species | slope of species | participant | species | Model fit was singular | | | |  |  |  |
| Set 2: addition of empathy to M6 | | | | | | | | | | | | |
| **Model specification** | **Model name** | **Fixed Effects** | **Fixed Effects added** | **Random intercept** | **Random slope** | **AIC** | **BIC** | **-2LL** | **df** | **Comparison** | **df** | **X^2^** |
| FE main effects + 2-way interaction + covariates | M9 | image size +  image brightness +  image pupil size +  species +  image pupil size × species +  empathy | empathy | participant |  | -3857.03 | -3763.25 | -3885.03 | 14 | M9 vs M6 | 1 | -1.22 |
| FE main effects + 2-way interactions + covariates | M10 | image size +  image brightness +  image pupil size +  species +  image pupil size × species +  empathy +  empathy × image pupil size | empathy × image pupil size | participant |  | -3855.02 | -3747.84 | -3887.02 | 16 | M10 vs M9 | 2 | -2.00 |
| FE main effects + 2-way interactions + covariates | M11 | image size +  image brightness +  image pupil size +  species +  image pupil size × species +  empathy +  empathy × species | empathy × species | participant |  | -3853.32 | -3746.15 | -3885.32 | 16 | M11 vs M9 | 2 | -0.30 |
| FE main effects + 2-way interactions + covariates | M12 | image size +  image brightness +  image pupil size +  species +  image pupil size × species +  empathy +  empathy × image pupil size +  empathy × species | empathy × image pupil size +  empathy × species | participant |  | -3851.31 | -3730.74 | -3887.31 | 18 | M12 vs M11 | 2 | -1.99 |
| FE main effects + 2-way interactions + 3-way interaction + covariates | M13 | image size +  image brightness +  image pupil size +  species +  image pupil size × species +  empathy +  empathy × image pupil size +  empathy × species +  empathy × image pupil size × species | empathy × image pupil size × species | participant |  | -3845.43 | -3698.06 | -3889.43 | 22 | M13 vs M12 | 4 | -2.11 |
| Set 3: addition of cat person/attitude to M6 | | | | | | | | | | | | |
| **Model specification** | **Model name** | **Fixed Effects** | **Fixed Effects added** | **Random intercept** | **Random slope** | **AIC** | **BIC** | **-2LL** | **df** | **Comparison** | **df** | **X^2^** |
| FE main effects + 2-way interaction + covariates | M14 | image size +  image brightness +  image pupil size +  species +  image pupil size × species +  cat person/attitude | cat person/attitude | participant |  | -3856.14 | -3762.37 | -3884.14 | 14 | M14 vs M6 | 1 | -0.34 |
| FE main effects + 2-way interactions + covariates | M15 | image size +  image brightness +  image pupil size +  species +  image pupil size × species +  cat person/attitude +  cat person/attitude × image pupil size | cat person/attitude × image pupil size | participant |  | -3852.73 | -3745.56 | -3884.74 | 16 | M15 vs M14 | 2 | -0.60 |
| FE main effects + 2-way interactions + covariates | M16 | image size +  image brightness +  image pupil size +  species +  image pupil size × species +  cat person/attitude +  cat person/attitude × species | cat person/attitude × species | participant |  | -3853.15 | -3745.97 | -3885.14 | 16 | M16 vs M14 | 2 | -1.00 |
| FE main effects + 2-way interactions + covariates | M17 | image size +  image brightness +  image pupil size +  species +  image pupil size × species +  cat person/attitude +  cat person/attitude × image pupil size +  cat person/attitude × species | cat person/attitude × image pupil size +  cat person/attitude × species | participant |  | -3849.73 | -3729.16 | -3885.74 | 18 | M17 vs M16 | 2 | -0.60 |
| FE main effects + 2-way interactions + 3-way interaction + covariates | M18 | image size +  image brightness +  image pupil size +  species +  image pupil size × species +  cat person/attitude +  cat person/attitude × image pupil size +  cat person/attitude × species +  cat person/attitude × image pupil size × species | cat person/attitude × image pupil size × species | participant |  | -3842.75 | -3695.38 | -3886.74 | 22 | M18 vs M17 | 4 | -1.00 |
| Set 4: addition of dog person/attitude to M6 | | | | | | | | | | | | |
| **Model specification** | **Model name** | **Fixed Effects** | **Fixed Effects added** | **Random intercept** | **Random slope** | **AIC** | **BIC** | **-2LL** | **df** | **Comparison** | **df** | **X^2^** |
| FE main effects + 2-way interaction + covariates | M19 | image size +  image brightness +  image pupil size +  species +  image pupil size × species +  dog person/attitude | dog person/attitude | participant |  | -3855.82 | -3762.04 | -3883.82 | 14 | M19 vs M6 | 1 | -0.02 |
| FE main effects + 2-way interactions + covariates | M20 | image size +  image brightness +  image pupil size +  species +  image pupil size × species +  dog person/attitude +  dog person/attitude × image pupil size | dog person/attitude × image pupil size | participant |  | -3853.33 | -3746.16 | -3885.33 | 16 | M20 vs M19 | 2 | -1.52 |
| FE main effects + 2-way interactions + covariates | M21 | image size +  image brightness +  image pupil size +  species +  image pupil size × species +  dog person/attitude +  dog person/attitude × species | dog person/attitude × species | participant |  | -3860.42 | -3753.25 | -3892.42 | 16 | M21 vs M19 | 2 | -8.60* |
| FE main effects + 2-way interactions + covariates | M22 | image size +  image brightness +  image pupil size +  species +  image pupil size × species +  dog person/attitude +  dog person/attitude × image pupil size +  dog person/attitude × species | dog person/attitude × image pupil size +  dog person/attitude × species | participant |  | -3857.98 | -3737.41 | -3893.98 | 18 | M22 vs M21 | 2 | -1.56 |
| FE main effects + 2-way interactions + 3-way interaction + covariates | M23 | image size +  image brightness +  image pupil size +  species +  image pupil size × species +  dog person/attitude +  dog person/attitude × image pupil size +  dog person/attitude × species +  dog person/attitude × image pupil size × species | dog person/attitude × image pupil size × species | participant |  | -3857.40 | -3710.04 | -3901.40 | 22 | M23 vs M22 | 4 | -7.42 |
| Set 5: addition of lifetime number of pet cats (square root) to M6 | | | | | | | | | | | | |
| **Model specification** | **Model name** | **Fixed Effects** | **Fixed Effects added** | **Random intercept** | **Random slope** | **AIC** | **BIC** | **-2LL** | **df** | **Comparison** | **df** | **X^2^** |
| FE main effects + 2-way interaction + covariates | M24 | image size +  image brightness +  image pupil size +  species +  image pupil size × species +  number of cats | number of cats | participant |  | -3856.09 | -3762.32 | -3884.10 | 14 | M24 vs M6 | 1 | -0.30 |
| FE main effects + 2-way interactions + covariates | M25 | image size +  image brightness +  image pupil size +  species +  image pupil size × species +  number of cats +  number of cats × image pupil size | number of cats × image pupil size | participant |  | -3852.69 | -3745.52 | -3884.68 | 16 | M25 vs M24 | 2 | -0.58 |
| FE main effects + 2-way interactions + covariates | M26 | image size +  image brightness +  image pupil size +  species +  image pupil size × species +  number of cats +  number of cats × species | number of cats × species | participant |  | -3852.13 | -3744.95 | -3884.12 | 16 | M26 vs M24 | 2 | -0.02 |
| FE main effects + 2-way interactions + covariates | M27 | image size +  image brightness +  image pupil size +  species +  image pupil size × species +  number of cats +  number of cats × image pupil size +  number of cats × species | number of cats × image pupil size +  number of cats × species | participant |  | -3848.72 | -3728.15 | -3884.72 | 18 | M27 vs M26 | 2 | -0.60 |
| FE main effects + 2-way interactions + 3-way interaction + covariates | M28 | image size +  image brightness +  image pupil size +  species +  image pupil size × species +  number of cats +  number of cats × image pupil size +  number of cats × species +  number of cats × image pupil size × species | number of cats × image pupil size × species | participant |  | -3841.94 | -3694.58 | -3885.94 | 22 | M28 vs M27 | 4 | -1.22 |
| Set 6: addition of lifetime number of pet dogs (square root) to M6 | | | | | | | | | | | | |
| **Model specification** | **Model name** | **Fixed Effects** | **Fixed Effects added** | **Random intercept** | **Random slope** | **AIC** | **BIC** | **-2LL** | **df** | **Comparison** | **df** | **X^2^** |
| FE main effects + 2-way interaction + covariates | M29 | image size +  image brightness +  image pupil size +  species +  image pupil size × species +  number of dogs | number of dogs | participant |  | -3856.40 | -3762.62 | -3884.40 | 14 | M29 vs M6 | 1 | -0.60 |
| FE main effects + 2-way interactions + covariates | M30 | image size +  image brightness +  image pupil size +  species +  image pupil size × species +  number of dogs +  number of dogs × image pupil size | number of dogs × image pupil size | participant |  | -3857.95 | -3750.78 | -3889.96 | 16 | M30 vs M29 | 2 | -5.56 |
| FE main effects + 2-way interactions + covariates | M31 | image size +  image brightness +  image pupil size +  species +  image pupil size × species +  number of dogs +  number of dogs × species | number of dogs × species | participant |  | -3852.46 | -3745.28 | -3884.46 | 16 | M31 vs M29 | 2 | -0.06 |
| FE main effects + 2-way interactions + covariates | M32 | image size +  image brightness +  image pupil size +  species +  image pupil size × species +  number of dogs +  number of dogs × image pupil size +  number of dogs × species | number of dogs × image pupil size +  number of dogs × species | participant |  | -3854.00 | -3733.43 | -3890.00 | 18 | M32 vs M31 | 2 | -5.54 |
| FE main effects + 2-way interactions + 3-way interaction + covariates | M33 | image size +  image brightness +  image pupil size +  species +  image pupil size × species +  number of dogs +  number of dogs × image pupil size +  number of dogs × species +  number of dogs × image pupil size × species | number of dogs × image pupil size × species | participant |  | -3850.01 | -3702.65 | -3894.02 | 22 | M33 vs M32 | 4 | -4.02 |

# TABLE B1

# *MODEL 1’S (NULL MODEL) FIXED EFFECT PARAMETER ESTIMATES AND RANDOM EFFECT VARIANCE AND CORRELATION VALUES*

| \|  \| \| \| \| \| \| \| \| \| \| \| \| \| \| \| \| \| --- \| --- \| --- \| --- \| --- \| --- \| --- \| --- \| --- \| --- \| --- \| --- \| --- \| --- \| --- \| --- \| \| **Fixed Effects Parameter Estimates** \| \| \| \| \| \| 95% Confidence Interval \| \| \| \|  \| \| \| \| \| \| \| Names \| \| Estimate \| \| *SE* \| \| Lower \| \| Upper \| \| *df* \| \| *t* \| \| *p* \| \| \| (Intercept) \|  \| 0.043 \|  \| 0.006 \|  \| 0.031 \|  \| 0.055 \|  \| 56.097 \|  \| 6.890 \|  \| < .001 \|  \| \|  \| \| \| \| \| \| \| \| \| \| \| \| \| \| \| \| |
| --- | --- | --- | --- | --- | --- | --- | --- | --- | --- | --- | --- | --- | --- | --- | --- | --- | --- | --- | --- | --- | --- | --- | --- | --- | --- | --- | --- | --- | --- | --- | --- | --- | --- | --- | --- | --- | --- | --- | --- | --- | --- | --- | --- | --- | --- | --- | --- | --- | --- | --- | --- | --- | --- | --- | --- | --- | --- | --- | --- | --- | --- | --- | --- | --- | --- | --- | --- | --- | --- | --- | --- | --- | --- | --- | --- | --- | --- | --- | --- | --- |
| \| **Random Components** \| Groups \| \| Name \| \| *SD* \| \| Variance \| \| *ICC* \| \| \| --- \| --- \| --- \| --- \| --- \| --- \| --- \| --- \| --- \| --- \| --- \| \|  \| Participant \|  \| (Intercept) \|  \| 0.042 \|  \| 0.002 \|  \| 0.035 \|  \| \|  \| Residual \|  \|  \|  \| 0.220 \|  \| 0.048 \|  \|  \|  \| |

ICC = intraclass correlation

# TABLE B2

# *MODEL 2’S FIXED EFFECT PARAMETER ESTIMATES AND RANDOM EFFECT VARIANCE AND CORRELATION VALUES*

| \|  \| \| \| \| \| \| \| \| \| \| \| \| \| \| \| \| \| --- \| --- \| --- \| --- \| --- \| --- \| --- \| --- \| --- \| --- \| --- \| --- \| --- \| --- \| --- \| --- \| \| **Fixed Effects Parameter Estimates** \| \| \| \| \| \| 95% Confidence Interval \| \| \| \|  \| \| \| \| \| \| \| Names \| \| Estimate \| \| *SE* \| \| Lower \| \| Upper \| \| *df* \| \| *t* \| \| *p* \| \| \| (Intercept) \|  \| 0.043 \|  \| 0.006 \|  \| 0.031 \|  \| 0.055 \|  \| 56.092 \|  \| 6.872 \|  \| < .001 \|  \| \| image_size \|  \| 0.001 \|  \| 9.230e-5 \|  \| 9.175e-4 \|  \| 0.001 \|  \| 5938.201 \|  \| 11.900 \|  \| < .001 \|  \| |
| --- | --- | --- | --- | --- | --- | --- | --- | --- | --- | --- | --- | --- | --- | --- | --- | --- | --- | --- | --- | --- | --- | --- | --- | --- | --- | --- | --- | --- | --- | --- | --- | --- | --- | --- | --- | --- | --- | --- | --- | --- | --- | --- | --- | --- | --- | --- | --- | --- | --- | --- | --- | --- | --- | --- | --- | --- | --- | --- | --- | --- | --- | --- | --- | --- | --- | --- | --- | --- | --- | --- | --- | --- | --- | --- | --- | --- | --- | --- | --- | --- |
| \| **Random Components** \| Groups \| \| Name \| \| *SD* \| \| Variance \| \| *ICC* \| \| \| --- \| --- \| --- \| --- \| --- \| --- \| --- \| --- \| --- \| --- \| --- \| \|  \| Participant \|  \| (Intercept) \|  \| 0.042 \|  \| 0.002 \|  \| 0.036 \|  \| \|  \| Residual \|  \|  \|  \| 0.217 \|  \| 0.047 \|  \|  \|  \| |

# TABLE B3

# *MODEL 3’S FIXED EFFECT PARAMETER ESTIMATES AND RANDOM EFFECT VARIANCE AND CORRELATION VALUES*

| \| **Fixed Effects Parameter Estimates** \| \| \| \| \| \| 95% Confidence Interval \| \| \| \|  \| \| \| \| \| \| \| --- \| --- \| --- \| --- \| --- \| --- \| --- \| --- \| --- \| --- \| --- \| --- \| --- \| --- \| --- \| --- \| \| Names \| \| Estimate \| \| *SE* \| \| Lower \| \| Upper \| \| *df* \| \| *t* \| \| *p* \| \| \| (Intercept) \|  \| 0.043 \|  \| 0.006 \|  \| 0.031 \|  \| 0.055 \|  \| 56.054 \|  \| 6.905 \|  \| < .001 \|  \| \| image_size \|  \| 0.001 \|  \| 7.448e-5 \|  \| 9.060e-4 \|  \| 0.001 \|  \| 5937.804 \|  \| 14.125 \|  \| < .001 \|  \| \| image_brightness \|  \| -0.008 \|  \| 1.430e-4 \|  \| -0.008 \|  \| -0.008 \|  \| 5938.258 \|  \| -56.414 \|  \| < .001 \|  \| |
| --- | --- | --- | --- | --- | --- | --- | --- | --- | --- | --- | --- | --- | --- | --- | --- | --- | --- | --- | --- | --- | --- | --- | --- | --- | --- | --- | --- | --- | --- | --- | --- | --- | --- | --- | --- | --- | --- | --- | --- | --- | --- | --- | --- | --- | --- | --- | --- | --- | --- | --- | --- | --- | --- | --- | --- | --- | --- | --- | --- | --- | --- | --- | --- | --- | --- | --- | --- | --- | --- | --- | --- | --- | --- | --- | --- | --- | --- | --- | --- | --- |
| \| **Random Components** \| Groups \| \| Name \| \| *SD* \| \| Variance \| \| *ICC* \| \| \| --- \| --- \| --- \| --- \| --- \| --- \| --- \| --- \| --- \| --- \| --- \| \|  \| Participant \|  \| (Intercept) \|  \| 0.043 \|  \| 0.002 \|  \| 0.058 \|  \| \|  \| Residual \|  \|  \|  \| 0.175 \|  \| 0.031 \|  \|  \|  \| |

# TABLE C1

# *MODEL 4’S FIXED EFFECT PARAMETER ESTIMATES AND RANDOM EFFECT VARIANCE AND CORRELATION VALUES*

| \| **Fixed Effects Parameter Estimates** \| \| \| \| \| \| \| \| 95% Confidence Interval \| \| \| \|  \| \| \| \| \| \| \| --- \| --- \| --- \| --- \| --- \| --- \| --- \| --- \| --- \| --- \| --- \| --- \| --- \| --- \| --- \| --- \| --- \| --- \| \| Names \| \| Effect \| \| Estimate \| \| *SE* \| \| Lower \| \| Upper \| \| *df* \| \| *t* \| \| *p* \| \| \| (Intercept) \|  \| (Intercept) \|  \| 0.043 \|  \| 0.006 \|  \| 0.031 \|  \| 0.055 \|  \| 56.054 \|  \| 6.898 \|  \| < .001 \|  \| \| image_size \|  \| image_size \|  \| 0.001 \|  \| 7.440e-5 \|  \| 9.048e-4 \|  \| 0.001 \|  \| 5937.800 \|  \| 14.121 \|  \| < .001 \|  \| \| image_brightness \|  \| image_brightness \|  \| -0.008 \|  \| 1.429e-4 \|  \| -0.008 \|  \| -0.008 \|  \| 5938.254 \|  \| -56.475 \|  \| < .001 \|  \| \| image_pupil_size1 \|  \| linear \|  \| -0.014 \|  \| 0.004 \|  \| -0.022 \|  \| -0.006 \|  \| 5937.154 \|  \| -3.593 \|  \| < .001 \|  \| \| image_pupil_size2 \|  \| quadratic \|  \| 0.002 \|  \| 0.004 \|  \| -0.005 \|  \| 0.010 \|  \| 5937.182 \|  \| 0.616 \|  \| 0.538 \|  \| \|  \| \| \| \| \| \| \| \| \| \| \| \| \| \| \| \| \| \| |
| --- | --- | --- | --- | --- | --- | --- | --- | --- | --- | --- | --- | --- | --- | --- | --- | --- | --- | --- | --- | --- | --- | --- | --- | --- | --- | --- | --- | --- | --- | --- | --- | --- | --- | --- | --- | --- | --- | --- | --- | --- | --- | --- | --- | --- | --- | --- | --- | --- | --- | --- | --- | --- | --- | --- | --- | --- | --- | --- | --- | --- | --- | --- | --- | --- | --- | --- | --- | --- | --- | --- | --- | --- | --- | --- | --- | --- | --- | --- | --- | --- | --- | --- | --- | --- | --- | --- | --- | --- | --- | --- | --- | --- | --- | --- | --- | --- | --- | --- | --- | --- | --- | --- | --- | --- | --- | --- | --- | --- | --- | --- | --- | --- | --- | --- | --- | --- | --- | --- | --- | --- | --- | --- | --- | --- | --- | --- | --- | --- | --- | --- | --- | --- | --- | --- | --- | --- | --- | --- | --- | --- | --- | --- | --- | --- |
| \| **Random Components** \| Groups \| \| Name \| \| *SD* \| \| Variance \| \| *ICC* \| \| \| --- \| --- \| --- \| --- \| --- \| --- \| --- \| --- \| --- \| --- \| --- \| \|  \| Participant \|  \| (Intercept) \|  \| 0.043 \|  \| 0.002 \|  \| 0.058 \|  \| \|  \| Residual \|  \|  \|  \| 0.175 \|  \| 0.031 \|  \|  \|  \| |

# TABLE C2

# *MODEL 5’S FIXED EFFECT PARAMETER ESTIMATES AND RANDOM EFFECT VARIANCE AND CORRELATION VALUES*

| \| **Fixed Effects Parameter Estimates** \| \| \| \| \| \| \| \| 95% Confidence Interval \| \| \| \|  \| \| \| \| \| \| \| --- \| --- \| --- \| --- \| --- \| --- \| --- \| --- \| --- \| --- \| --- \| --- \| --- \| --- \| --- \| --- \| --- \| --- \| \| Names \| \| Effect \| \| Estimate \| \| *SE* \| \| Lower \| \| Upper \| \| *df* \| \| *t* \| \| *p* \| \| \| (Intercept) \|  \| (Intercept) \|  \| 0.043 \|  \| 0.006 \|  \| 0.031 \|  \| 0.055 \|  \| 56.052 \|  \| 6.872 \|  \| < .001 \|  \| \| image_size \|  \| image_size \|  \| -0.002 \|  \| 2.728e-4 \|  \| -0.002 \|  \| -0.001 \|  \| 5939.447 \|  \| -5.916 \|  \| < .001 \|  \| \| image_brightness \|  \| image_brightness \|  \| -0.008 \|  \| 1.479e-4 \|  \| -0.008 \|  \| -0.007 \|  \| 5937.542 \|  \| -51.739 \|  \| < .001 \|  \| \| image_pupil_size1 \|  \| linear \|  \| -0.015 \|  \| 0.004 \|  \| -0.023 \|  \| -0.008 \|  \| 5937.169 \|  \| -3.959 \|  \| < .001 \|  \| \| image_pupil_size2 \|  \| quadratic \|  \| 0.002 \|  \| 0.004 \|  \| -0.006 \|  \| 0.009 \|  \| 5937.164 \|  \| 0.468 \|  \| 0.640 \|  \| \| species1 \|  \| cat - human \|  \| 0.015 \|  \| 0.005 \|  \| 0.005 \|  \| 0.026 \|  \| 5937.877 \|  \| 2.818 \|  \| 0.005 \|  \| \| species2 \|  \| dog - human \|  \| 0.187 \|  \| 0.018 \|  \| 0.152 \|  \| 0.222 \|  \| 5939.163 \|  \| 10.469 \|  \| < .001 \|  \| |
| --- | --- | --- | --- | --- | --- | --- | --- | --- | --- | --- | --- | --- | --- | --- | --- | --- | --- | --- | --- | --- | --- | --- | --- | --- | --- | --- | --- | --- | --- | --- | --- | --- | --- | --- | --- | --- | --- | --- | --- | --- | --- | --- | --- | --- | --- | --- | --- | --- | --- | --- | --- | --- | --- | --- | --- | --- | --- | --- | --- | --- | --- | --- | --- | --- | --- | --- | --- | --- | --- | --- | --- | --- | --- | --- | --- | --- | --- | --- | --- | --- | --- | --- | --- | --- | --- | --- | --- | --- | --- | --- | --- | --- | --- | --- | --- | --- | --- | --- | --- | --- | --- | --- | --- | --- | --- | --- | --- | --- | --- | --- | --- | --- | --- | --- | --- | --- | --- | --- | --- | --- | --- | --- | --- | --- | --- | --- | --- | --- | --- | --- | --- | --- | --- | --- | --- | --- | --- | --- | --- | --- | --- | --- | --- | --- | --- | --- | --- | --- | --- | --- | --- | --- | --- | --- | --- | --- | --- | --- | --- | --- | --- | --- |
| \| **Random Components** \| Groups \| \| Name \| \| *SD* \| \| Variance \| \| *ICC* \| \| \| --- \| --- \| --- \| --- \| --- \| --- \| --- \| --- \| --- \| --- \| --- \| \|  \| Participant \|  \| (Intercept) \|  \| 0.044 \|  \| 0.002 \|  \| 0.059 \|  \| \|  \| Residual \|  \|  \|  \| 0.173 \|  \| 0.030 \|  \|  \|  \| |
